# Tetrel bond in the triphenyltin(IV) chloride–cyclo­hexyl­diphenyl­phosphane oxide (1/1) cocrystal

**DOI:** 10.1107/S2414314623006375

**Published:** 2023-08-01

**Authors:** Sachin Liyanage, Jeffrey S. Ovens, David L. Bryce

**Affiliations:** a Department of Chemistry and Biomolecular Sciences, University of Ottawa, Ottawa, Ontario, K1N6N5, Canada; Howard University, USA

**Keywords:** crystal structure, tetrel bond, noncovalent inter­action, cocrystal

## Abstract

The single-crystal X-ray diffraction structure of the title compound, [SnCl(C_6_H_5_)_3_]·C_18_H_21_OP, is reported. The 1:1 cocrystal features a short and directional tetrel bond between tin and oxygen. The tin–oxygen distance is 2.346 (4) Å, representing 62% of the sum of the van der Waals radii of Sn and O. The Cl—Sn⋯O angle is 174.0 (1)° and this nearly linear arrangement is consistent with a tetrel bond formed *via* a σ-hole opposite the tin–chlorine covalent bond. Some weak C—H⋯Cl inter­actions are noted between adjacent mol­ecules.

## Abstract 2

A short and directional tetrel bond between tin and oxygen is identified in the title compound.

## Graphical abstract

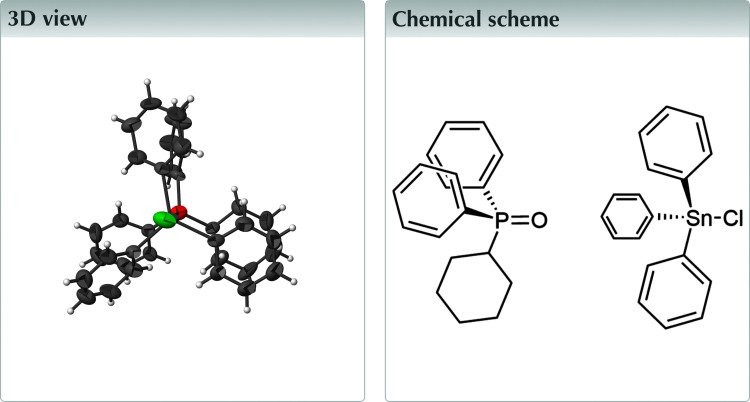

## Structure description

**Figure 1:**
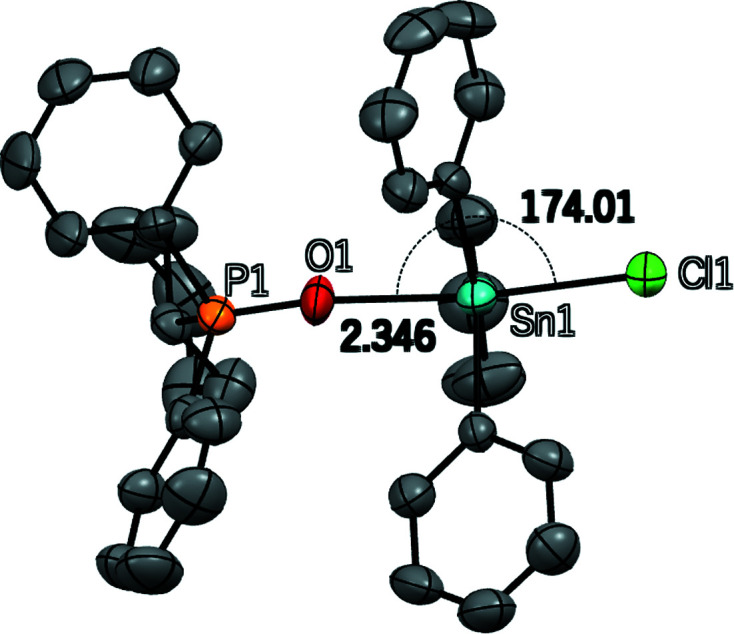
The mol­ecular structure of the title compound. The tin–oxygen tetrel bond distance and chlorine–tin–oxygen angle are shown. H atoms are not shown. H atoms have been omitted for clarity.

**Figure 2:**
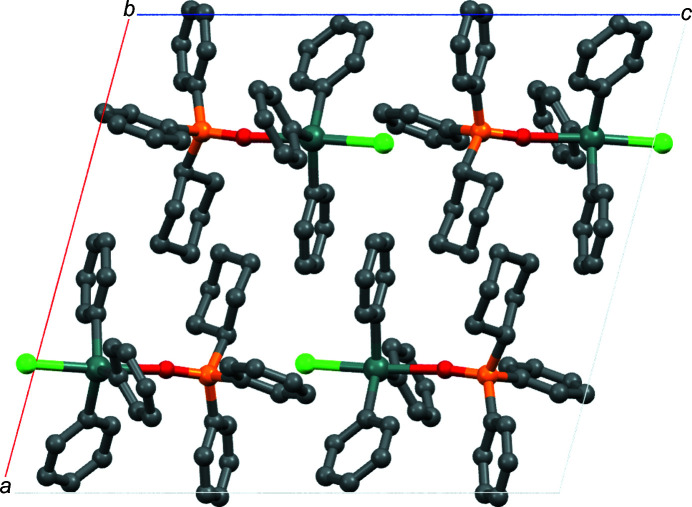
Packing diagram of the title compound, viewed along the *b* axis. H atoms have been omitted for clarity.

The tetrel bond (TB), a moderately strong and directional noncovalent inter­action, has received renewed inter­est in recent years as a useful structure-directing element and crystal engineering tool (Bauzá *et al.*, 2016 ▸). TBs form between a region of depleted electron density and elevated electrostatic potential (σ-hole) on a Group 14 (tetrel) element and an electron-donor moiety. Scilabra *et al.* (2018 ▸) have reviewed the literature and summarized the available information on TBs involving tin and germanium. The title compound features a short and highly linear TB between the Sn^IV^ atom of triphenyl­tin(IV) chloride and the O atom of cyclo­hexyl­diphenyl­phosphane oxide. The asymmetric unit consists of one complete mol­ecule of each type. The tin–oxygen distance is 2.346 (4) Å and the Cl—Sn⋯O TB angle is 174.0 (1)° (Fig. 1 ▸). This distance represents approximately 62% of the sum of the van der Waals radii of Sn and O. The nearly linear arrangement is consistent with a TB inter­action *via* a σ-hole opposite the tin–chlorine covalent bond. These metrics may be compared to those for an analogous system comprised of tri­methyl­tin chloride and tri­phenyl­phosphane oxide, where the tin–oxygen TB distance is 2.375 (2) Å and the Cl—Sn⋯O TB angle is 177.57 (7)° (Davis *et al.*, 2007 ▸). Similar metrics are reported for the tin–oxygen TBs in [chlorido­bis­(*p*-chloro­phen­yl)(*p*-tol­yl)tin]-μ-1,2-bis­(di­phenyl­phosphor­yl)ethane-κ^2^ *O*:*O*′-[bromido­bis­(*p*-chloro­phen­yl)(*p*-tol­yl)tin] (Lo & Ng, 2004 ▸), (Ph_2_ClSnCH_2_)_2_·(Me_2_N)_2_PO (Jurkschat *et al.*, 1990 ▸) and bromido­tri(*p*-tol­yl)tin–hexa­methyl­phospho­r­amide (Lo *et al.*, 2001 ▸), and for a series of cocrystals of SnPPh_3_Cl formed with pyridine *N*-oxides, di­methyl­urea, and di­phenyl sulfoxide (Kumar *et al.*, 2020 ▸). The packing of the title compound (Fig. 2 ▸) does not feature any other strong noncovalent inter­actions; the only other weak inter­actions of note are between the Cl atom and the H atoms of the phenyl rings of adjacent mol­ecules (Table 1 ▸).

## Synthesis and crystallization

In a typical procedure, triphenyltin(IV) chloride (0.0614 g) and cyclo­hexyl­diphenyl­phosphane (0.0894 g) were added to hexane (60 ml) in a beaker. The mixture was heated and stirred until the solids were completely dissolved. Cocrystals grew *via* slow evaporation of the solvent in a fume hood over a period of 5 d. Evidently, during the synthesis, the phosphane was oxidized to give the phosphane oxide, as the process was not carried out under an inert atmosphere.

## Refinement

The crystal data, data collection and structure refinement details are summarized in Table 2 ▸. H atoms were placed geometrically and refined using a riding model.

## Supplementary Material

Crystal structure: contains datablock(s) I. DOI: 10.1107/S2414314623006375/bv4048sup1.cif

Structure factors: contains datablock(s) I. DOI: 10.1107/S2414314623006375/bv4048Isup2.hkl

Supporting information file. DOI: 10.1107/S2414314623006375/bv4048Isup3.cml

CCDC reference: 2267964

Additional supporting information:  crystallographic information; 3D view; checkCIF report

## Figures and Tables

**Table 1:** Hydrogen-bond geometry (Å, °)

*D*—H⋯*A*	*D*—H	H⋯*A*	*D*⋯*A*	*D*—H⋯*A*
C1—H1⋯Cl1^i^	0.98	2.87	3.803 (6)	159
C18—H18⋯Cl1^i^	0.93	2.88	3.637 (7)	139

Symmetry code: (i) 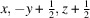 .

**Table 2:** Experimental details

Crystal data	
Chemical formula	[SnCl(C_6_H_5_)_3_]·C_18_H_21_OP
*M*_r_	669.76
Crystal system, space group	Monoclinic, *P*2_1_/*c*
Temperature (K)	273
*a*, *b*, *c* (Å)	16.548 (2), 10.7496 (15), 18.665 (3)
β (°)	105.110 (4)
*V* (Å^3^)	3205.4 (8)
*Z*	4
Radiation type	Mo *K*α
μ (mm^−1^)	0.96
Crystal size (mm)	0.31 × 0.17 × 0.08

Data collection	
Diffractometer	Bruker APEXII CCD
Absorption correction	Multi-scan (*SADABS*; Sheldrick, 1996 ▸)
*T*_min_, *T*_max_	0.621, 0.745
No. of measured, independent and observed [*I* > 2σ(*I*)] reflections	19012, 5547, 2948
*R*_int_	0.090
(sin θ/λ)_max_ (Å^−1^)	0.598

Refinement	
*R*[*F* ^2^ > 2σ(*F* ^2^)], *wR*(*F* ^2^), *S*	0.052, 0.116, 0.95
No. of reflections	5547
No. of parameters	361
H-atom treatment	H-atom parameters constrained
Δρ_max_, Δρ_min_ (e Å^−3^)	1.08, −0.62

Computer programs: *APEX3* (Bruker, 2012 ▸), *SAINT* (Bruker, 2012 ▸), *SHELXT2014* (Sheldrick, 2015*a* ▸) and *SHELXL2018* (Sheldrick, 2015*b* ▸).

## full crystallographic data

### Crystal data

**Table:**

[SnCl(C_6_H_5_)_3_]·C_18_H_21_OP	*F*(000) = 1368
*M**_r_* = 669.76	*D*_x_ = 1.388 Mg m^−^^3^
Monoclinic, *P*2_1_/*c*	Mo *K*α radiation, λ = 0.71073 Å
*a* = 16.548 (2) Å	Cell parameters from 4222 reflections
*b* = 10.7496 (15) Å	θ = 2.3–21.9°
*c* = 18.665 (3) Å	µ = 0.96 mm^−^^1^
β = 105.110 (4)°	*T* = 273 K
*V* = 3205.4 (8) Å^3^	Plate, colourless
*Z* = 4	0.31 × 0.17 × 0.08 mm

### Data collection

**Table:**

Bruker APEXII CCD diffractometer	2948 reflections with *I* > 2σ(*I*)
Graphite monochromator	*R*_int_ = 0.090
ω and πhi scans	θ_max_ = 25.1°, θ_min_ = 1.3°
Absorption correction: multi-scan (SADABS; Sheldrick, 1996)	*h* = −19→13
*T*_min_ = 0.621, *T*_max_ = 0.745	*k* = −12→12
19012 measured reflections	*l* = −22→21
5547 independent reflections	

### Refinement

**Table:**

Refinement on *F*^2^	Primary atom site location: structure-invariant direct methods
Least-squares matrix: full	Hydrogen site location: inferred from neighbouring sites
*R*[*F*^2^ > 2σ(*F*^2^)] = 0.052	H-atom parameters constrained
*wR*(*F*^2^) = 0.116	*w* = 1/[σ^2^(*F*_o_^2^) + (0.0456*P*)^2^] where *P* = (*F*_o_^2^ + 2*F*_c_^2^)/3
*S* = 0.95	(Δ/σ)_max_ = 0.001
5547 reflections	Δρ_max_ = 1.08 e Å^−^^3^
361 parameters	Δρ_min_ = −0.62 e Å^−^^3^
0 restraints	

### Special details

**Table:**

Geometry. All esds (except the esd in the dihedral angle between two l.s. planes) are estimated using the full covariance matrix. The cell esds are taken into account individually in the estimation of esds in distances, angles and torsion angles; correlations between esds in cell parameters are only used when they are defined by crystal symmetry. An approximate (isotropic) treatment of cell esds is used for estimating esds involving l.s. planes.
Refinement. Crystallographic data for the title compound were collected from a single crystal mounted on a MiTeGen MicroMount using parabar oil. Data were collected on a Bruker Kappa APEXII single-crystal diffractometer equipped with a sealed tube Mo Kα source (λ = 0.71073 Å), a TRIUMPH monochromator, and an APEXII CCD detector. Data were collected at 273 K. Raw data collection and processing were performed with the APEX3 software package from Bruker. Initial unit-cell parameters were determined from 36 data frames from select ω scans. Semi-empirical absorption corrections based on equivalent reflections were applied. Systematic absences in the diffraction data set and unit-cell parameters were consistent with the assigned space group. The initial structural solutions were determined using SHELXT (Sheldrick, 2015b) direct methods, and refined with full-matrix least-squares procedures based on F^2^ using SHELXT and ShelXle. The structure was deposited with the Cambridge Structural Database, entry 2267964.

### Fractional atomic coordinates and isotropic or equivalent isotropic displacement parameters (Å^2^)

**Table:**

	*x*	*y*	*z*	*U*_iso_*/*U*_eq_	
Sn1	0.74139 (3)	0.42607 (4)	0.61347 (3)	0.03965 (17)	
Cl1	0.73072 (12)	0.49797 (15)	0.48345 (10)	0.0583 (5)	
P1	0.75522 (10)	0.33662 (14)	0.81497 (10)	0.0371 (4)	
O1	0.7432 (2)	0.3773 (3)	0.7364 (2)	0.0453 (11)	
C1	0.6708 (4)	0.2398 (5)	0.8277 (3)	0.0405 (17)	
H1	0.689087	0.199472	0.876358	0.049*	
C2	0.6492 (4)	0.1379 (6)	0.7680 (4)	0.066 (2)	
H2A	0.634718	0.176145	0.719269	0.079*	
H2AB	0.697817	0.085500	0.771633	0.079*	
C3	0.5761 (5)	0.0578 (7)	0.7768 (5)	0.084 (3)	
H3A	0.561586	−0.001785	0.736459	0.101*	
H3AB	0.593000	0.011609	0.822989	0.101*	
C4	0.4999 (5)	0.1360 (8)	0.7770 (5)	0.093 (3)	
H4A	0.478894	0.174469	0.728660	0.111*	
H4AB	0.456136	0.082823	0.785875	0.111*	
C5	0.5204 (5)	0.2359 (7)	0.8358 (5)	0.084 (3)	
H5A	0.535447	0.197608	0.884526	0.101*	
H5AB	0.471428	0.287535	0.832419	0.101*	
C6	0.5928 (4)	0.3169 (6)	0.8262 (4)	0.068 (2)	
H6A	0.575657	0.360988	0.779322	0.081*	
H6AB	0.606258	0.378255	0.865633	0.081*	
C7	0.7615 (4)	0.4664 (5)	0.8759 (4)	0.0374 (16)	
C8	0.7922 (5)	0.4522 (6)	0.9517 (4)	0.064 (2)	
H8	0.811506	0.375067	0.971498	0.077*	
C9	0.7940 (5)	0.5540 (8)	0.9986 (4)	0.081 (3)	
H9	0.811615	0.543443	1.049801	0.097*	
C10	0.7702 (5)	0.6682 (7)	0.9697 (5)	0.074 (3)	
H10	0.774125	0.736428	1.001112	0.089*	
C11	0.7409 (5)	0.6832 (6)	0.8954 (5)	0.064 (2)	
H11	0.723488	0.761391	0.876009	0.077*	
C12	0.7366 (4)	0.5823 (6)	0.8480 (4)	0.0486 (18)	
H12	0.716663	0.593431	0.796977	0.058*	
C13	0.8527 (4)	0.2548 (5)	0.8477 (3)	0.0407 (17)	
C14	0.9247 (5)	0.3158 (6)	0.8458 (4)	0.061 (2)	
H14	0.920827	0.397493	0.828876	0.074*	
C15	1.0024 (5)	0.2619 (8)	0.8677 (5)	0.076 (2)	
H15	1.049931	0.306512	0.865676	0.091*	
C16	1.0092 (6)	0.1404 (8)	0.8928 (5)	0.085 (3)	
H16	1.061254	0.102139	0.908556	0.102*	
C17	0.9372 (5)	0.0774 (7)	0.8939 (4)	0.075 (2)	
H17	0.940741	−0.005004	0.909617	0.090*	
C18	0.8603 (4)	0.1338 (6)	0.8723 (4)	0.0526 (19)	
H18	0.812718	0.089502	0.874382	0.063*	
C19	0.6260 (4)	0.3297 (6)	0.5840 (3)	0.0414 (17)	
C20	0.6255 (4)	0.2067 (7)	0.5648 (4)	0.070 (2)	
H20	0.675363	0.168679	0.562754	0.084*	
C21	0.5530 (5)	0.1384 (7)	0.5487 (5)	0.091 (3)	
H21	0.554667	0.054460	0.537100	0.109*	
C22	0.4785 (5)	0.1921 (8)	0.5494 (5)	0.080 (3)	
H22	0.429551	0.145177	0.538574	0.096*	
C23	0.4765 (5)	0.3139 (8)	0.5661 (4)	0.067 (2)	
H23	0.425880	0.352059	0.565347	0.081*	
C24	0.5496 (5)	0.3818 (6)	0.5843 (4)	0.063 (2)	
H24	0.547573	0.465132	0.597188	0.076*	
C25	0.7582 (4)	0.6126 (5)	0.6541 (4)	0.0381 (17)	
C26	0.6976 (4)	0.7008 (6)	0.6302 (4)	0.057 (2)	
H26	0.648295	0.679564	0.595325	0.068*	
C27	0.7085 (5)	0.8211 (6)	0.6573 (5)	0.070 (2)	
H27	0.666909	0.880293	0.640582	0.085*	
C28	0.7811 (6)	0.8529 (6)	0.7090 (5)	0.072 (3)	
H28	0.788339	0.933676	0.727340	0.087*	
C29	0.8420 (5)	0.7677 (7)	0.7334 (4)	0.069 (2)	
H29	0.891609	0.789705	0.767645	0.082*	
C30	0.8296 (5)	0.6455 (6)	0.7064 (4)	0.0529 (19)	
H30	0.870562	0.585861	0.724317	0.063*	
C31	0.8464 (4)	0.3065 (5)	0.6194 (3)	0.0375 (16)	
C32	0.8992 (4)	0.3271 (6)	0.5743 (4)	0.053 (2)	
H32	0.889843	0.395901	0.543017	0.064*	
C33	0.9659 (4)	0.2488 (7)	0.5739 (4)	0.065 (2)	
H33	1.000849	0.265544	0.543254	0.078*	
C34	0.9795 (5)	0.1462 (7)	0.6194 (5)	0.068 (2)	
H34	1.023708	0.092630	0.619618	0.081*	
C35	0.9280 (5)	0.1233 (6)	0.6643 (5)	0.068 (2)	
H35	0.937693	0.054158	0.695318	0.081*	
C36	0.8610 (4)	0.2022 (6)	0.6642 (4)	0.054 (2)	
H36	0.825900	0.184534	0.694567	0.065*	

### Atomic displacement parameters (Å^2^)

**Table:**

	*U*^11^	*U*^22^	*U*^33^	*U*^12^	*U*^13^	*U*^23^
Sn1	0.0529 (3)	0.0298 (2)	0.0381 (3)	0.0012 (2)	0.0154 (2)	−0.0019 (2)
Cl1	0.0890 (14)	0.0499 (11)	0.0404 (13)	0.0168 (10)	0.0248 (10)	0.0063 (8)
P1	0.0485 (11)	0.0284 (9)	0.0367 (12)	−0.0030 (8)	0.0152 (9)	0.0011 (8)
O1	0.065 (3)	0.047 (3)	0.028 (3)	−0.006 (2)	0.018 (2)	0.000 (2)
C1	0.048 (4)	0.032 (4)	0.040 (5)	−0.003 (3)	0.009 (3)	0.004 (3)
C2	0.078 (6)	0.061 (5)	0.063 (6)	−0.023 (4)	0.025 (5)	−0.011 (4)
C3	0.098 (7)	0.072 (6)	0.082 (7)	−0.052 (6)	0.021 (5)	−0.012 (5)
C4	0.069 (7)	0.097 (7)	0.103 (9)	−0.027 (6)	0.007 (6)	0.027 (6)
C5	0.050 (5)	0.074 (6)	0.131 (9)	−0.002 (5)	0.029 (5)	0.017 (6)
C6	0.054 (5)	0.056 (5)	0.096 (7)	0.003 (4)	0.025 (5)	0.002 (4)
C7	0.050 (4)	0.031 (4)	0.033 (5)	0.012 (3)	0.015 (3)	0.001 (3)
C8	0.101 (6)	0.051 (5)	0.036 (5)	0.017 (4)	0.008 (4)	−0.005 (4)
C9	0.128 (8)	0.079 (6)	0.028 (5)	0.018 (5)	0.005 (5)	−0.008 (4)
C10	0.115 (7)	0.048 (5)	0.064 (7)	0.009 (5)	0.030 (6)	−0.016 (5)
C11	0.095 (6)	0.043 (5)	0.064 (6)	0.002 (4)	0.037 (5)	−0.001 (4)
C12	0.060 (5)	0.043 (4)	0.046 (5)	0.000 (4)	0.020 (4)	−0.001 (4)
C13	0.046 (4)	0.038 (4)	0.043 (5)	−0.005 (3)	0.019 (3)	−0.002 (3)
C14	0.063 (5)	0.042 (4)	0.084 (7)	−0.005 (4)	0.027 (5)	0.000 (4)
C15	0.041 (5)	0.093 (7)	0.095 (7)	0.000 (5)	0.021 (5)	0.001 (5)
C16	0.069 (6)	0.083 (6)	0.112 (8)	0.031 (5)	0.039 (6)	0.017 (6)
C17	0.076 (6)	0.068 (5)	0.090 (7)	0.030 (5)	0.037 (5)	0.026 (5)
C18	0.052 (5)	0.048 (4)	0.066 (6)	0.004 (4)	0.030 (4)	0.008 (4)
C19	0.057 (5)	0.035 (4)	0.032 (4)	−0.002 (3)	0.010 (4)	−0.003 (3)
C20	0.045 (5)	0.065 (5)	0.092 (7)	−0.006 (4)	0.003 (4)	−0.038 (5)
C21	0.062 (6)	0.062 (5)	0.137 (9)	−0.010 (5)	0.003 (6)	−0.038 (5)
C22	0.058 (6)	0.075 (6)	0.095 (8)	−0.018 (5)	0.000 (5)	−0.021 (5)
C23	0.040 (5)	0.081 (6)	0.077 (7)	0.007 (5)	0.006 (4)	−0.009 (5)
C24	0.065 (6)	0.052 (5)	0.068 (6)	0.007 (4)	0.010 (5)	−0.012 (4)
C25	0.050 (5)	0.030 (4)	0.038 (5)	0.007 (3)	0.017 (4)	0.007 (3)
C26	0.062 (5)	0.051 (5)	0.057 (6)	−0.008 (4)	0.015 (4)	0.001 (4)
C27	0.074 (6)	0.035 (4)	0.108 (8)	0.015 (4)	0.032 (6)	0.007 (4)
C28	0.099 (7)	0.033 (4)	0.101 (8)	−0.014 (5)	0.054 (6)	−0.019 (5)
C29	0.083 (6)	0.050 (5)	0.071 (6)	−0.015 (5)	0.017 (5)	−0.015 (4)
C30	0.069 (5)	0.042 (4)	0.048 (5)	0.003 (4)	0.014 (4)	0.002 (4)
C31	0.047 (4)	0.034 (4)	0.032 (4)	0.000 (3)	0.011 (3)	0.002 (3)
C32	0.055 (5)	0.042 (4)	0.064 (6)	0.008 (4)	0.016 (4)	0.009 (4)
C33	0.056 (5)	0.072 (6)	0.071 (6)	0.003 (4)	0.024 (4)	0.003 (5)
C34	0.069 (6)	0.061 (5)	0.074 (7)	0.020 (5)	0.020 (5)	−0.006 (5)
C35	0.087 (7)	0.047 (5)	0.063 (6)	0.027 (5)	0.009 (5)	0.004 (4)
C36	0.068 (5)	0.047 (4)	0.050 (5)	0.005 (4)	0.021 (4)	−0.001 (4)

### Geometric parameters (Å, º)

**Table:**

Sn1—C19	2.115 (6)	C15—C16	1.382 (10)
Sn1—C25	2.136 (6)	C15—H15	0.9300
Sn1—C31	2.141 (6)	C16—C17	1.375 (10)
Sn1—O1	2.346 (4)	C16—H16	0.9300
Sn1—Cl1	2.5089 (18)	C17—C18	1.372 (8)
P1—O1	1.493 (4)	C17—H17	0.9300
P1—C7	1.786 (6)	C18—H18	0.9300
P1—C13	1.798 (6)	C19—C20	1.370 (8)
P1—C1	1.807 (6)	C19—C24	1.384 (8)
C1—C6	1.527 (8)	C20—C21	1.371 (9)
C1—C2	1.537 (8)	C20—H20	0.9300
C1—H1	0.9800	C21—C22	1.366 (10)
C2—C3	1.528 (9)	C21—H21	0.9300
C2—H2A	0.9700	C22—C23	1.348 (9)
C2—H2AB	0.9700	C22—H22	0.9300
C3—C4	1.516 (10)	C23—C24	1.377 (9)
C3—H3A	0.9700	C23—H23	0.9300
C3—H3AB	0.9700	C24—H24	0.9300
C4—C5	1.510 (10)	C25—C26	1.367 (8)
C4—H4A	0.9700	C25—C30	1.369 (8)
C4—H4AB	0.9700	C26—C27	1.383 (9)
C5—C6	1.530 (9)	C26—H26	0.9300
C5—H5A	0.9700	C27—C28	1.373 (10)
C5—H5AB	0.9700	C27—H27	0.9300
C6—H6A	0.9700	C28—C29	1.350 (9)
C6—H6AB	0.9700	C28—H28	0.9300
C7—C12	1.372 (8)	C29—C30	1.402 (8)
C7—C8	1.382 (8)	C29—H29	0.9300
C8—C9	1.396 (9)	C30—H30	0.9300
C8—H8	0.9300	C31—C32	1.381 (8)
C9—C10	1.358 (9)	C31—C36	1.382 (8)
C9—H9	0.9300	C32—C33	1.390 (8)
C10—C11	1.355 (9)	C32—H32	0.9300
C10—H10	0.9300	C33—C34	1.374 (9)
C11—C12	1.390 (8)	C33—H33	0.9300
C11—H11	0.9300	C34—C35	1.364 (10)
C12—H12	0.9300	C34—H34	0.9300
C13—C14	1.369 (8)	C35—C36	1.397 (9)
C13—C18	1.375 (8)	C35—H35	0.9300
C14—C15	1.372 (9)	C36—H36	0.9300
C14—H14	0.9300		
			
C19—Sn1—C25	125.2 (3)	C14—C13—P1	117.9 (5)
C19—Sn1—C31	112.6 (2)	C18—C13—P1	125.0 (5)
C25—Sn1—C31	121.1 (2)	C13—C14—C15	123.0 (7)
C19—Sn1—O1	85.74 (19)	C13—C14—H14	118.5
C25—Sn1—O1	83.99 (19)	C15—C14—H14	118.5
C31—Sn1—O1	90.7 (2)	C14—C15—C16	119.2 (8)
C19—Sn1—Cl1	93.90 (17)	C14—C15—H15	120.4
C25—Sn1—Cl1	91.37 (17)	C16—C15—H15	120.4
C31—Sn1—Cl1	94.95 (18)	C17—C16—C15	118.4 (8)
O1—Sn1—Cl1	174.01 (10)	C17—C16—H16	120.8
O1—P1—C7	111.5 (3)	C15—C16—H16	120.8
O1—P1—C13	110.8 (3)	C18—C17—C16	121.3 (7)
C7—P1—C13	105.5 (3)	C18—C17—H17	119.4
O1—P1—C1	112.9 (3)	C16—C17—H17	119.4
C7—P1—C1	106.6 (3)	C17—C18—C13	121.0 (7)
C13—P1—C1	109.2 (3)	C17—C18—H18	119.5
P1—O1—Sn1	172.1 (3)	C13—C18—H18	119.5
C6—C1—C2	109.7 (5)	C20—C19—C24	116.7 (6)
C6—C1—P1	111.3 (4)	C20—C19—Sn1	119.0 (5)
C2—C1—P1	110.9 (5)	C24—C19—Sn1	124.4 (5)
C6—C1—H1	108.3	C19—C20—C21	121.3 (7)
C2—C1—H1	108.3	C19—C20—H20	119.3
P1—C1—H1	108.3	C21—C20—H20	119.3
C3—C2—C1	111.4 (6)	C22—C21—C20	120.8 (7)
C3—C2—H2A	109.4	C22—C21—H21	119.6
C1—C2—H2A	109.4	C20—C21—H21	119.6
C3—C2—H2AB	109.4	C23—C22—C21	119.2 (7)
C1—C2—H2AB	109.4	C23—C22—H22	120.4
H2A—C2—H2AB	108.0	C21—C22—H22	120.4
C4—C3—C2	111.7 (7)	C22—C23—C24	120.0 (7)
C4—C3—H3A	109.3	C22—C23—H23	120.0
C2—C3—H3A	109.3	C24—C23—H23	120.0
C4—C3—H3AB	109.3	C23—C24—C19	121.9 (6)
C2—C3—H3AB	109.3	C23—C24—H24	119.0
H3A—C3—H3AB	107.9	C19—C24—H24	119.0
C5—C4—C3	111.5 (7)	C26—C25—C30	118.5 (6)
C5—C4—H4A	109.3	C26—C25—Sn1	121.3 (5)
C3—C4—H4A	109.3	C30—C25—Sn1	120.2 (5)
C5—C4—H4AB	109.3	C25—C26—C27	120.9 (7)
C3—C4—H4AB	109.3	C25—C26—H26	119.5
H4A—C4—H4AB	108.0	C27—C26—H26	119.5
C4—C5—C6	110.7 (7)	C28—C27—C26	119.8 (7)
C4—C5—H5A	109.5	C28—C27—H27	120.1
C6—C5—H5A	109.5	C26—C27—H27	120.1
C4—C5—H5AB	109.5	C29—C28—C27	120.5 (7)
C6—C5—H5AB	109.5	C29—C28—H28	119.7
H5A—C5—H5AB	108.1	C27—C28—H28	119.7
C1—C6—C5	111.9 (5)	C28—C29—C30	119.2 (7)
C1—C6—H6A	109.2	C28—C29—H29	120.4
C5—C6—H6A	109.2	C30—C29—H29	120.4
C1—C6—H6AB	109.2	C25—C30—C29	121.1 (7)
C5—C6—H6AB	109.2	C25—C30—H30	119.4
H6A—C6—H6AB	107.9	C29—C30—H30	119.4
C12—C7—C8	118.9 (6)	C32—C31—C36	117.4 (6)
C12—C7—P1	120.4 (5)	C32—C31—Sn1	120.5 (5)
C8—C7—P1	120.7 (5)	C36—C31—Sn1	122.0 (5)
C7—C8—C9	119.9 (7)	C31—C32—C33	122.5 (6)
C7—C8—H8	120.1	C31—C32—H32	118.8
C9—C8—H8	120.1	C33—C32—H32	118.8
C10—C9—C8	120.2 (7)	C34—C33—C32	119.0 (7)
C10—C9—H9	119.9	C34—C33—H33	120.5
C8—C9—H9	119.9	C32—C33—H33	120.5
C11—C10—C9	120.3 (7)	C35—C34—C33	119.8 (7)
C11—C10—H10	119.9	C35—C34—H34	120.1
C9—C10—H10	119.9	C33—C34—H34	120.1
C10—C11—C12	120.3 (7)	C34—C35—C36	120.9 (7)
C10—C11—H11	119.9	C34—C35—H35	119.6
C12—C11—H11	119.9	C36—C35—H35	119.6
C7—C12—C11	120.4 (7)	C31—C36—C35	120.4 (7)
C7—C12—H12	119.8	C31—C36—H36	119.8
C11—C12—H12	119.8	C35—C36—H36	119.8
C14—C13—C18	117.1 (6)		
			
O1—P1—C1—C6	−75.5 (5)	C1—P1—C13—C18	5.3 (7)
C7—P1—C1—C6	47.2 (5)	C18—C13—C14—C15	−0.6 (11)
C13—P1—C1—C6	160.7 (5)	P1—C13—C14—C15	−178.5 (6)
O1—P1—C1—C2	46.9 (5)	C13—C14—C15—C16	0.2 (12)
C7—P1—C1—C2	169.7 (5)	C14—C15—C16—C17	0.8 (13)
C13—P1—C1—C2	−76.8 (5)	C15—C16—C17—C18	−1.4 (13)
C6—C1—C2—C3	−54.9 (8)	C16—C17—C18—C13	1.0 (12)
P1—C1—C2—C3	−178.3 (5)	C14—C13—C18—C17	0.0 (10)
C1—C2—C3—C4	55.1 (9)	P1—C13—C18—C17	177.7 (5)
C2—C3—C4—C5	−55.3 (9)	C24—C19—C20—C21	−1.6 (11)
C3—C4—C5—C6	55.6 (9)	Sn1—C19—C20—C21	176.9 (6)
C2—C1—C6—C5	56.0 (8)	C19—C20—C21—C22	1.6 (13)
P1—C1—C6—C5	179.2 (5)	C20—C21—C22—C23	0.2 (14)
C4—C5—C6—C1	−56.7 (9)	C21—C22—C23—C24	−1.9 (13)
O1—P1—C7—C12	15.1 (6)	C22—C23—C24—C19	2.0 (12)
C13—P1—C7—C12	135.5 (5)	C20—C19—C24—C23	−0.2 (11)
C1—P1—C7—C12	−108.5 (6)	Sn1—C19—C24—C23	−178.6 (5)
O1—P1—C7—C8	−164.4 (5)	C30—C25—C26—C27	1.0 (10)
C13—P1—C7—C8	−44.0 (6)	Sn1—C25—C26—C27	179.4 (5)
C1—P1—C7—C8	72.0 (6)	C25—C26—C27—C28	−0.1 (12)
C12—C7—C8—C9	2.6 (11)	C26—C27—C28—C29	0.3 (13)
P1—C7—C8—C9	−177.9 (6)	C27—C28—C29—C30	−1.3 (12)
C7—C8—C9—C10	−3.7 (13)	C26—C25—C30—C29	−2.1 (11)
C8—C9—C10—C11	3.1 (13)	Sn1—C25—C30—C29	179.6 (5)
C9—C10—C11—C12	−1.4 (13)	C28—C29—C30—C25	2.2 (11)
C8—C7—C12—C11	−1.0 (10)	C36—C31—C32—C33	−1.2 (10)
P1—C7—C12—C11	179.5 (5)	Sn1—C31—C32—C33	−177.1 (5)
C10—C11—C12—C7	0.4 (11)	C31—C32—C33—C34	0.7 (11)
O1—P1—C13—C14	58.0 (6)	C32—C33—C34—C35	−0.3 (12)
C7—P1—C13—C14	−62.8 (6)	C33—C34—C35—C36	0.5 (12)
C1—P1—C13—C14	−177.0 (5)	C32—C31—C36—C35	1.3 (10)
O1—P1—C13—C18	−119.7 (6)	Sn1—C31—C36—C35	177.2 (5)
C7—P1—C13—C18	119.5 (6)	C34—C35—C36—C31	−1.0 (11)

### Hydrogen-bond geometry (Å, º)

**Table:**

*D*—H···*A*	*D*—H	H···*A*	*D*···*A*	*D*—H···*A*
C1—H1···Cl1^i^	0.98	2.87	3.803 (6)	159
C18—H18···Cl1^i^	0.93	2.88	3.637 (7)	139

Symmetry code: (i) *x*, −*y*+1/2, *z*+1/2.
